# Associations of *PPARG* and *PPARGC1A* Polymorphisms with Ritodrine-Induced Adverse Events in Patients with Preterm Labor

**DOI:** 10.3390/jpm15050212

**Published:** 2025-05-21

**Authors:** Eun Jeong Jang, Da Hoon Lee, Yubin Song, Jung Sun Kim, Young Ju Kim, Jeong Yee, Hye Sun Gwak

**Affiliations:** 1 College of Pharmacy and Graduate School of Pharmaceutical Sciences, Ewha Womans University, Seoul 03760, Republic of Korea; heimdall01@hanmail.net (E.J.J.); hoon0515@ewha.ac.kr (D.H.L.); yubinsong1130@gmail.com (Y.S.); 2 Department of Pharmacy, Kangwon National University, Chuncheon 24341, Republic of Korea; jungsun.kim@kangwon.ac.kr; 3 Department of Obstetrics and Gynecology, School of Medicine, Ewha Womans University, Seoul 07985, Republic of Korea; kkyj@ewha.ac.kr; 4 School of Pharmacy, Sungkyunkwan University, Suwon 16419, Republic of Korea; j.yee@skku.edu

**Keywords:** ritodrine, adverse events, peroxisome proliferator-activated receptor gamma, *PPARG*, *PPARGC1A*, gene polymorphism

## Abstract

**Objectives:** Ritodrine, a tocolytic agent used to delay preterm labor, can cause several cardiovascular-associated adverse events (AEs). This study aimed to examine the relationship between gene polymorphisms in peroxisome proliferator-activated receptor gamma (*PPARG*) and PPARG coactivator-1α (*PPARGC1A*) and the occurrence of ritodrine-induced AEs. Additionally, a risk-scoring system was developed to identify patients at high risk of AEs. **Methods:** Patients aged 18 years or older who were administered ritodrine to manage preterm labor with intact membranes and uterine contractions occurring at 20–36 weeks of gestation were enrolled in this study. A total of 70 common *PPARG* and *PPARGC1A* variants (minor allele frequency ≥ 0.2) with low linkage disequilibrium (r^2^ < 0.8) were selected from an Axiom™ Precision Medicine Research Array (AMPRA). **Results:** A total of 149 patients were included in the analysis. After adjusting for confounders (age, gestational age, and the maximum infusion rate), weight and rs2946385, rs35523565, and rs2240748 of *PPARGC1A* were identified as significant predictors associated with ritodrine-induced AEs. Based on the risk-scoring system, the predicted probabilities of AEs for patients with scores of 0, 1, 2, 3, 4, and 5 points were 4%, 9%, 18%, 35%, 55%, and 74%, respectively. The AUROC for the risk score predicting ritodrine-induced AEs was 0.729 (95% CI: 0.672–0.831, *p* < 0.001). **Conclusions:** This study indicates that ritodrine-induced AEs are related to *PPARGC1A* polymorphisms. A risk-scoring system based on genetic variants showed moderate predictive ability for ritodrine-induced AEs, suggesting potential utility in females with preterm labor.

## 1. Introduction

Preterm birth is labor occurring at less than 37 weeks of gestation, which can lead to neonatal morbidity and mortality and has significant social and economic consequences for health systems [1,2]. The World Health Organization reported that the global preterm birth rate is around 10%, with over 13.4 million babies born prematurely in 2020 [3].

Several tocolytic medications are used to prevent preterm labor by suppressing uterine contractions and extending the duration of a pregnancy, allowing for further fetal development, particularly lung maturation, which is crucial for the survival and health of a fetus [4,5]. Among these medications, beta-2-adrenergic receptor (ADRB2) agonists such as ritodrine act by stimulating ADRB2 in various tissues, including uterine smooth muscle cells, leading to muscle relaxation and suppression of contractions [6]. While ritodrine was once a standard treatment for delaying preterm labor, its potential for adverse events (AEs) has led to the adoption of safer alternatives in the United States [7]. Ritodrine-induced AEs include tachycardia, palpitations, tremors, chest pain, and pulmonary edema [8,9]. The known risk factors for ritodrine-induced AEs are advanced maternal age, multiple pregnancies, and concurrent cardiovascular disease [10]. In addition, genetic factors were shown to impact ritodrine-induced AEs in prior studies. For example, polymorphisms in the *ADRB2* gene, the regulator of a G-protein signaling gene, and the Gs subunit gene have been associated with ritodrine-induced AEs [11,12].

Peroxisome proliferator-activated receptor gamma (PPARG) and PPARG coactivator-1α (PPARGC1A) play vital roles in regulating the gene expression involved in various metabolic processes, particularly those related to glucose metabolism, lipid metabolism, and adipogenesis [13]. PPARG functions as a crucial transcription factor that regulates cardiovascular health through multiple mechanisms: improving metabolic flexibility, suppressing inflammation, reducing fibrosis, and modulating vascular pathways [14,15]. PPARGC1A regulates these activities by functioning as PPARG’s principal coactivator [16]. Since the predominant adverse effects of ritodrine—tachycardia, palpitations, and chest pain—are cardiovascular in nature, there may be a mechanistic connection between these genes and ritodrine-induced AEs. Several studies have shown that genetic variations in *PPARG* and *PPARGC1A* are related to cardiovascular conditions [17,18]. For example, Pro12Ala of *PPARG* (rs1801282) plays a significant role in cardiovascular health through its influence on lipid metabolism, inflammatory responses, and blood pressure regulation [19], whereas Gly482Ser of *PPARGC1A* (rs8192678) has been studied in connection with cardiovascular conditions, including coronary artery disease and hypertension [20,21].

In addition to their well-established roles in cardiovascular regulation, PPARG and PPARGC1A have been increasingly recognized as key modulators of pharmacological responses across a diverse range of drug classes. For instance, meta-analyses have reported that specific *PPARG* genotypes are significantly associated with interindividual variation in thiazolidinedione efficacy, particularly with respect to glycemic control markers such as hemoglobin A1c, fasting plasma glucose, and triglyceride levels [22]. *PPARGC1A* variants have been shown to influence therapeutic responses to rosiglitazone in diabetic populations [23]. Moreover, evidence suggests that *PPARGC1A* polymorphisms can alter the metabolic effects of metformin, particularly in the regulation of triacylglycerol levels [24]. Beyond antidiabetic agents, *PPARG* haplotypes have also been linked to heterogeneous lipid-lowering responses following fluvastatin therapy, indicating a broader role in lipid metabolism [25]. Additionally, recent research identified interactive associations between *PPARG* and *PPARGC1A* polymorphisms and bisphosphonate-related osteonecrosis of the jaw in patients with osteoporosis [26].

Since *PPARG* and *PPARGC1A* are associated with the efficacy and adverse effects of various medications, both may be connected to ritodrine-induced AEs. However, direct interaction between ritodrine and *PPARG* or *PPARGC1A* has not yet been established. Therefore, the objective of this research was to explore the genetic links between *PPARG* and *PPARGC1A* and the occurrence of ritodrine-induced AEs in pregnant females experiencing preterm labor.

## 2. Methods

### 2.1. Study Population

We conducted this prospective investigation at Ewha Womans University Mokdong Hospital between January 2010 and October 2016. The research protocol received approval from the hospital’s Institutional Review Board (IRB No. 217-1-26) and adhered to the guidelines of the Declaration of Helsinki. Each participant enrolled in this study provided written informed consent before inclusion. Participants were initially enrolled if they met the following clinical criteria: aged 18 years or older, a diagnosis of preterm labor (20–36 weeks of gestation) with intact membranes, and uterine activity defined as at least three contractions within ten minutes accompanied by observable cervical changes. Cervical changes were assessed via digital vaginal examination by board-certified obstetricians. Antepartum digital cervical examination has traditionally been performed using the Bishop score, which is calculated based on an assessment of cervical dilatation, effacement, consistency, position, and fetal station [27]. In this study, a modified Bishop score was calculated using the sum of the cervical dilatation and effacement scores to objectively evaluate the cervical status at enrollment.

Prior to analysis, patients were excluded based on several predefined criteria to minimize confounding factors and ensure the reliability of the results. Patients who received ritodrine as a pre-treatment for McDonald’s cervical cerclage were excluded, as the indication and timing of drug administration differed from those for preterm labor management. Patients presenting with urgent obstetric complications requiring immediate delivery (e.g., fetal distress, pre-eclampsia, severe oligohydramnios, placenta previa, placental abruption, or spontaneous premature rupture of membranes) were excluded to avoid the influence of acute clinical conditions that could independently affect outcomes or preclude sustained ritodrine administration. Those with comorbidities such as cardiovascular disease, hyperthyroidism, or asthma were excluded due to known contraindications to ritodrine and potential risks of AEs. Finally, patients were also excluded if adequate blood samples were unavailable, as they were essential for downstream pharmacogenetic analysis.

### 2.2. Drug Administration

Ritodrine (Lavopa^®^, JW Pharmaceutical, Seoul, Republic of Korea) was administered intravenously, starting at an initial dose of 0.05 mg/min and increasing by 0.05 mg/min every ten minutes until the desired uterine response was reached. Once the desired uterine response was reached, maintenance therapy was continued at a rate of 0.05 mg/min.

### 2.3. Data Collection and Outcomes

Demographic information on maternal age, the gestational age at the start of ritodrine administration, weight, body mass index (BMI), the maximum infusion rate, and the modified Bishop score was extracted from paper and electronic medical records using the BESTcare 2.0 (ezCaretech, Seoul, Republic of Korea) program. The primary outcome was AEs induced by ritodrine, defined as tachycardia (≥100 beats per minute), palpitations, shortness of breath, dyspnea, or pulmonary edema that necessitated either a dosage reduction or complete discontinuation of the medication.

### 2.4. Genotyping

Genotyping was conducted using an Axiom^™^ Precision Medicine Research Array (Thermo Fisher Scientific, Waltham, MA, USA) following genomic DNA extraction, which was performed using peripheral blood samples with a QIAamp DNA Blood Mini Kit (QIAGEN, Hilden, Germany). The genotyping procedures were conducted prior to outcome analysis without knowledge of patient status. A total of 70 *PPARG* and *PPARGC1A* single-nucleotide polymorphisms (SNPs) were selected from the AMPRA based on the linkage disequilibrium (LD) (r^2^ < 0.8) and minor allele frequency (MAF) (MAF ≥ 0.2) in the Asian population. SNPs with low call rates (<90%) or significant deviation from Hardy–Weinberg equilibrium (*p* < 1 × 10^−5^) were also excluded. Genetic information was curated from the National Center for Biotechnology Information and Haploreg 4.1 [28,29].

### 2.5. Statistical Analyses

To compare patients who experienced ritodrine-induced AEs with those who did not, categorical variables were analyzed using either the chi-squared test or Fisher’s exact test, while continuous variables were evaluated with independent *t*-tests. For each SNP, both dominant and recessive models were applied, and the model demonstrating the strongest association—based on the effect size and statistical significance—was selected. To identify independent predictors of ritodrine-related AEs, logistic regression was performed, incorporating variables that showed significance in univariate analysis (*p* < 0.05), along with clinically relevant factors such as age, gestational age, and the peak infusion rate. A multivariable logistic model was then used to construct a scoring system where each variable’s adjusted odds ratio (AOR) was normalized relative to the smallest AOR in the model and rounded to the nearest whole number. An area under the receiver operating curve (AUROC) analysis was performed to assess the performance of the model. The predicted risk and observed risk were then calculated. All statistical analyses were conducted using SPSS version 20 (IBM, Chicago, IL, USA), and a *p*-value lower than 0.05 was considered statistically significant.

## 3. Results

Out of the 216 participants initially enrolled, 67 were excluded because of the use of ritodrine as a premedication for McDonald’s surgery (*n* = 10), severe conditions resulting in preterm labor prior to hospital admission (*n* = 14), existing cardiovascular diseases (*n* = 7), and insufficient blood samples (*n* = 36). Consequently, 149 individuals remained eligible for the final analysis.

Table 1 lists the baseline characteristics of the enrolled participants. The average maternal age was 30.9 ± 3.7 years, and the mean gestational age at the initiation of drug therapy was 29.6 ± 3.7 weeks. The average weight and BMI were 62.5 ± 8.4 kg and 24.1 ± 3.0 kg/m^2^, respectively. Among the demographics, the weight of the patients was statistically significantly related to AEs, with patients weighing less than 60 kg having a higher risk of AEs. The mean maximum infusion rate in the AE group was over 10 mL/h higher compared with the non-AE group; however, the difference between the two groups was marginally significant.

Table 2 shows the genotypes significantly associated with ritodrine-induced AEs among the SNPs studied (Supplementary Table S1). Among the SNPs analyzed, rs6808179 of *PPARG* and rs2946385, rs35523565, rs2240748, and rs10002521 of *PPARGC1A* were significantly associated with ritodrine-induced AEs.

After adjusting for confounders (age, gestational age, and the maximum infusion rate), the weight of the patient and rs2946385, rs35523565, and rs2240748 of *PPARGC1A* were identified as significant predictors associated with ritodrine-induced AEs (Table 3). Patients with a weight below 60 kg had a 2.99-fold (95% CI: 1.26–7.04) higher risk of experiencing AEs than those weighing 60 kg or more. The *PPARGC1A* rs35523565 CT/TT genotypes were associated with the highest risk of AEs (AOR: 4.68, 95% CI: 1.76–12.44). Carriers of the *PPARGC1A* rs2946385 TT genotype and the rs2240748 GA/AA genotypes had 4.07-fold (95% CI: 1.43–11.58) and 3.27-fold (95% CI: 1.36–7.87) increased risks of AEs, respectively, compared with the non-carriers of each SNP.

**Figure 1:**
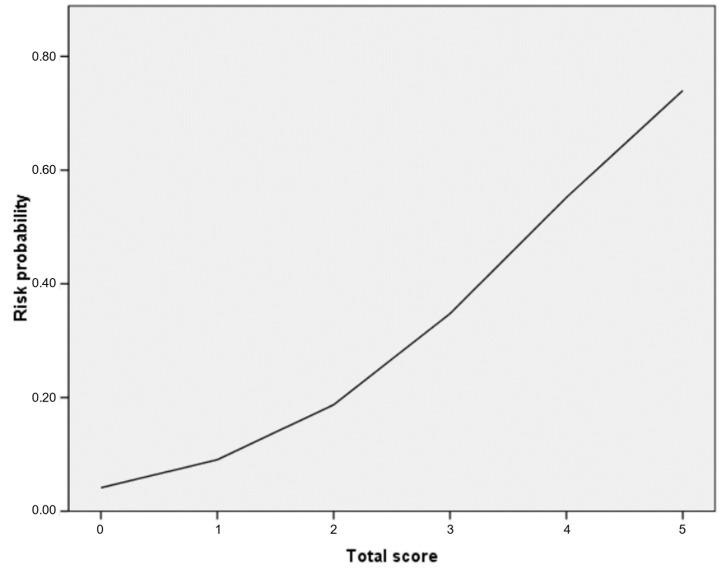
The logistic regression curve of the probability of ritodrine-induced adverse events.

**Figure 2:**
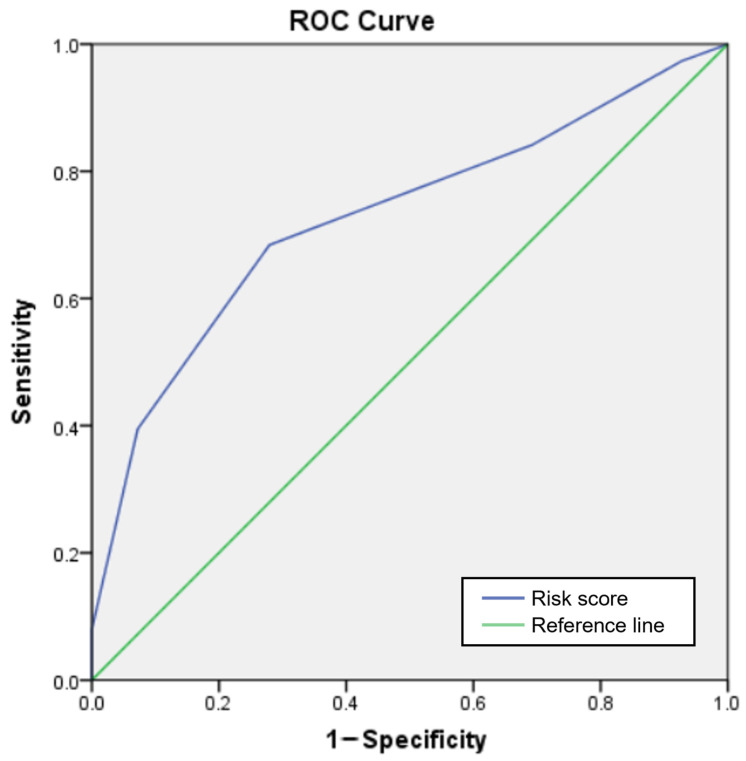
The area under the receiver operating curve (AUROC) of the risk score for ritodrine-induced adverse events.

Based on the significant predictors identified in the multivariable analysis, a scoring system was developed to estimate the risk of ritodrine-induced AEs (Table 4). The AOR of a weight lower than 60 kg, which was the smallest value among the variables, was used to standardize the AOR of each variable, and the values were rounded to the nearest integer. One point was assigned for a weight lower than 60 kg, the rs2946385 TT genotype, and the rs2240748 GA/AA genotype, while two points were assigned for the rs35523565 CT/TT genotype. The risk score varied from 0 to 5. The calculated probabilities of experiencing AEs for patients with scores of 0, 1, 2, 3, 4, and 5 were 11%, 16%, 13%, 32%, 60%, and 100%. The risk of ritodrine-induced AEs based on the logistic regression curve is shown in Figure 1. The predicted probabilities corresponding to risk scores of 0, 1, 2, 3, 4, and 5 were 4%, 9%, 18%, 35%, 55%, and 74%, respectively. The AUROC of the risk score for ritodrine-induced AEs was 0.729 (95% CI: 0.672–0.831, *p* < 0.001) (Figure 2).

## 4. Discussion

This research identified significant associations between weight and rs2946385, rs35523565, and rs2240748 in the *PPARGC1A* gene and the occurrence of ritodrine-induced AEs after controlling for confounding factors. The risk-scoring system demonstrated that higher scores correlated with an increased probability of experiencing ritodrine-induced AEs.

*PPARG* is abundantly expressed in immune cells, muscle tissue, liver tissue, and adipose tissue [13]. A study by Silva et al. demonstrated that an endothelial *PPARG* gene deficiency hastened age-related vascular dysfunction, inflammation, and cell aging through mechanisms involving elevated nicotinamide adenine dinucleotide phosphate (NADPH) oxidase and increased Rho-associated protein kinase activity [30]. In a previous study, a cardiomyocyte-specific *PPARG* deficiency in mice resulted in cardiac hypertrophy with preserved systolic function, primarily due to oxidative stress linked to mitochondrial damage, and this overexpression ultimately progressed to cardiac hypertrophy, dilation, and dysfunction within a few months [31,32]. *PPARG* rs6808179 demonstrated a significant association with AEs in the univariate analysis. A GTEx analysis identified the variant allele (G) of this SNP as a significant expression quantitative trait locus (eQTL) for the *PPARG* transcript that is associated with higher PPARG expression levels in the brain in the cerebellum and cerebellar hemisphere, with *p*-values of 6.6 × 10^−13^ and 1.8 × 10^−8^, respectively [33]. However, this SNP did not show a significant association with AEs in the multivariable analysis.

*PPARGC1A* is mostly expressed in skeletal muscle, brown adipose tissue, the heart, the liver, and the brain [34]. PPARGC1A contributes to cellular oxidative balance by regulating the expression of key antioxidant enzymes, including manganese superoxide dismutase and catalase. It plays an essential role in mitochondrial function and energy metabolism. Impaired activity of PPARGC1A has been linked to elevated oxidative stress and inflammation, which may underlie the development of various metabolic and cardiovascular diseases [35]. As a result, the regulation of oxidative stress and lipid metabolism by *PPARGC1A* may contribute to the onset and progression of atherosclerosis, ultimately leading to cardiovascular disease [36]. *PPARGC1A* rs2946385 was related to an approximately four-fold increase in the risk of ritodrine-induced AEs in this study. Rs2946385, which is one of the synonymous variants of *PPARGC1A*, has been reported to be associated with several diseases. Sharma et al. showed that rs2946385 was associated with an increased risk of type 2 diabetes [37]. In a study by Kim et al., individuals who were homozygous for the G allele of *PPARGC1A* rs2946385 had a 3.03-fold elevated risk of developing bisphosphonate-related osteonecrosis of the jaw compared with those with other genotypes after adjusting for confounders (95% CI: 1.01– 9.11) [26]. These findings suggest that this SNP may affect biological processes involved in a range of disease conditions. However, studies on rs35523565 and rs2240748 are limited, necessitating further research.

Among the demographic characteristics, only weight was related to ritodrine-induced AEs. Lower weights might result in more AEs because of higher drug concentrations in smaller patients. The relationship between weight and drug concentrations is important for optimizing the safety and efficacy of medications. Lower body weights can decrease the volume for distribution, leading to higher blood drug concentrations in individuals when given the same dose [38].

The limitations of this study include that it had a single-center design and involved only Asian females. Additionally, no formal sample size calculation was performed, and due to the small sample size, rare variants were not investigated. These factors may reduce the statistical power to detect modest associations. Furthermore, a multiple testing correction such as the Bonferroni adjustment was not applied, as an overly conservative correction could have increased the risk of false negatives in this hypothesis-driven study setting. Given the moderate AUROC observed, the predictive model requires further validation in larger and ethnically diverse populations. Also, the biological mechanisms were not examined. Therefore, further research is needed to elucidate the roles of *PPARG* and *PPARGC1A* gene polymorphisms in ritodrine-induced AEs and to validate their clinical utility.

## 5. Conclusions

This study explored whether *PPARG* and *PPARGC1A* gene polymorphisms are associated with AEs related to ritodrine administration in patients with preterm labor. Upon further validation, these findings and the developed risk-scoring system could aid in predicting the likelihood of ritodrine-induced AEs, thus facilitating more personalized treatments for these patients. Future research should focus on exploring the molecular mechanisms behind these associations and assessing the clinical applicability of genetic risk stratification in diverse populations.

## Figures and Tables

**Table 1:** Effects of demographic characteristics on ritodrine-induced adverse events.

Characteristic	Total	Adverse Event	No Adverse Event	*p*-Value
	(*n* = 149)	(*n* = 38)	(*n* = 111)	
Age (years)				0.555
<35	126 (84.6%)	31 (81.6%)	95 (85.6%)	
≥35	23 (15.4%)	7 (18.4%)	16 (14.4%)	
Gestational age (weeks)				0.470
<30	67(45.0%)	19 (50.0%)	48 (43.2%)	
≥30	82 (55.0%)	19 (50.0%)	63 (56.8%)	
Weight (kg)				0.008
<60	59 (39.6%)	22 (57.9%)	37 (33.3%)	
≥60	90 (60.4%)	16 (42.1%)	74 (66.7%)	
Body mass index (kg/m^2^)				0.515
<23	60 (40.3%)	17 (44.7%)	43 (38.7%)	
≥23	89 (59.7%)	21 (55.3%)	68 (61.3%)	
Maximum infusion rate (mL/h)	60.0 ± 33.6	68.7 ± 38.4	57.1 ± 31.3	0.067
Modified Bishop score ^a^				0.431
<2	78 (52.3%)	22 (73.3%)	57 (65.5%)	
≥2	69 (47.7%)	8 (26.7%)	30 (34.5%)	

^a^ The modified Bishop score is the sum of the dilation and effacement scores. Dilation scores: 0 = closed; 1 = 1 to 2 cm; 2 = 3 to 4 cm; 3 = 5+ cm. Effacement scores: 0 = 0 to 30%; 1 = 40 to 50%; 2 = 60 to 70%; 3 = 80+%.

**Table 2:** Effects of significant single-nucleotide polymorphisms in *PPARG* and *PPARGC1A* on ritodrine-induced adverse events.

Gene Polymorphism ^a^	Grouped Genotypes	Adverse Event	No Adverse Event	*p*-Value
		(*n* = 38)	(*n* = 111)	
*PPARG*				
rs6808179 (T > G)	TT	6 (15.8%)	38 (34.2%)	0.031
	TG, GG	32 (84.2%)	73 (65.8%)	
*PPARGC1A*				
rs2946385 (G > T)	GG, GT	27 (71.1%)	95 (88.0%)	0.016
	TT	11 (28.9%)	13 (12.0%)	
rs35523565 (C > T)	CC	9 (24.3%)	50 (45.0%)	0.026
	CT, TT	28 (75.7%)	61 (55.0%)	
rs2240748 (G > A)	GG	13 (35.1%)	64 (57.7%)	0.018
	GA, AA	24 (64.9%)	47 (42.3%)	
rs10002521 (C > T)	CC, CT	17 (44.7%)	71 (64.0%)	0.037
	TT	21 (55.3%)	40 (36.0%)	

*PPARG*, peroxisome proliferator-activated receptor-γ; *PPARGC1A*, PPARG coactivator-1α. ^a^ Significant SNPs (*p*-value < 0.05) among those listed in Supplementary Table S1.

**Table 3:** Multivariable analysis and risk-scoring system for ritodrine-induced adverse events.

Predictors	Unadjusted OR (95% CI)	Adjusted OR (95% CI)	*p*-Value	Score
Age (years) ≥ 35	1.34 (0.51–3.56)			
Gestational age (weeks) < 30	2.44 (0.62–9.62)			
Weight (kg) < 60	2.75 (1.29–5.85)	2.99 (1.26–7.04)	0.013	1
Maximum infusion rate (mL/h)	1.01 (1.00–1.02)			
*PPARG*				
rs6808179 TG/GG	2.78 (1.07–7.22)			
*PPARGC1A*				
rs2946385 TT	2.98 (1.20–7.39)	4.07 (1.43–11.58)	0.008	1
rs35523565 CT/TT	2.55 (1.10–5.90)	4.68 (1.76–12.44)	0.002	2
rs2240748 GA/AA	2.51 (1.16–5.45)	3.27 (1.36–7.87)	0.008	1
rs10002521 TT	2.19 (1.04–4.63)			

OR, odds ratio; *PPARG*, peroxisome proliferator-activated receptor-γ; *PPARGC1A*, PPARG coactivator-1α.

**Table 4:** The observed ritodrine-induced adverse events (%) and those predicted by the risk-scoring system.

Score	Patients with Adverse Events (*n*)	Total Patients (*n*)	Observed Adverse Event Risk (%)	Predicted Adverse Event Risk (%)
0	1	9	11.1	4.1
1	5	31	16.1	9.1
2	6	45	13.3	18.7
3	11	34	32.3	34.8
4	12	20	60.0	55.2
5	3	3	100.0	74.0

## Data Availability

The data that support the findings of this study are available from the corresponding author upon reasonable request.

## Supplementary Materials

The following supporting information can be downloaded at: https://www.mdpi.com/article/10.3390/jpm15050212/s1, Table S1: Demographic, clinical, and genetic characteristics of the study population.
